# Creative aging in virtual spaces: using museum content and music therapy to explore cultural diversity

**DOI:** 10.3389/fmed.2023.1273000

**Published:** 2023-11-30

**Authors:** Melita Belgrave, Katherine Palmer, Tana M. Luger Motyka

**Affiliations:** ^1^School of Music, Dance, and Theatre, Arizona State University, Tempe, AZ, United States; ^2^Musical Instrument Museum, Phoenix, AZ, United States; ^3^Covenant Health Network, Phoenix, AZ, United States

**Keywords:** creative aging, virtual programming, music therapy, museum, diversity

## Abstract

**Introduction:**

During the pandemic, many creative aging programs stopped being delivered in person, and practitioners turned to various virtual platforms to deliver content for older adults to maintain their cognitive, physical, and psychosocial well-being. Collaborators from a university-based music therapy program and a global music museum developed asynchronous virtual programs, one for wellness populations and another for memory care settings. Content was developed and delivered by the paper’s principal investigators in collaboration with the museum’s curatorial team and an upper division music therapy class composed of juniors and first-year graduate equivalency students (*n* = 21). The asynchronous program included museum gallery content and music therapy interventions of singing, movement, and/or instrument playing based on highlighted geographic regions. The purpose of the study was to explore older adults’ experiences when participating in the program.

**Methods:**

Fifty-six older adults from three post-acute care facilities (two skilled nursing facilities and one assisted living center) served as participants. Older adult participants were categorized as cognitively healthy (*n* = 27) or those diagnosed with dementia (*n* = 29) and attended five music sessions over 8 weeks, ranging from 30 to 60 min in length. A within-subject repeated measures design was used to investigate the impact of the creative aging program on older adults’ psychosocial well-being and engagement behaviors. Psychosocial well-being for cognitively healthy older adults were measured with the Multicultural Quality of Life Index, Engagement in Meaningful Activity Survey, and the PROMIS Social Isolation Short Form-4a. Psychosocial well-being for older adults with memory loss was measured with the Quality of Life in Late-Stage Dementia tool.

**Results:**

Cognitively healthy older adults showed an increase in psychological/emotional wellness after participating in the program, while older adults with memory loss appeared less irritable and physically uncomfortable and seemed to enjoy interacting with others more. Surprisingly, the cognitively healthy older adults also showed an increase in social isolation between the start and end of the program, which may not be related to the intervention, but to the fact that all three sites had COVID outbreaks during the study and had to pause their group activities programming and residents were required to stay in their rooms. Additionally, the music interventions fostered engagement behaviors of interest (facial expression, posture), and response (body movement, eye contact, and musical interaction with the leaders in the videos) for both groups of older adults. Instrument interventions were most engaging for cognitively healthy older adults. Singing interventions were most engaging for older adults with dementia, whereas movement interventions were less engaging for older adults with dementia.

**Discussion:**

Findings suggest that creative aging virtual programs can be delivered in asynchronous settings to enhance older adults’ well-being and foster engagement. Additionally, virtual programming may be used to augment ongoing programming or used to reach older adults when distance is a factor to enhance older adults’ well-being.

## Introduction

Creative aging is a field of study that aims to promote evidence-based healthful aging through continued engagement with artful practices (1). When developed with research-driven methodology, creative aging programs have been shown to provide cognitive, physical, and psychosocial benefits to older adults across many settings (2, 3). These programs are sometimes led by teaching artists in visual arts, music, dance, and literacy. The teaching artists develop interactive programs that allow older adults to experience art making throughout the various forms, join in group discussions, or attend performances and gallery openings. Sometimes these programs are attached to community centers, museums, and other arts agencies and healthcare organizations.

The topic of museums as places of engagement and socialization for older adults has become increasingly prevalent in aging research (4–7), which has resulted, in part, with health care professionals using museums as social prescription interventions (8). Research findings related to socially prescribed museum attendance note improvements in psychological well-being and potential long-term outcomes of sustained social capital and enhanced physical health (9). A 2017 analysis by the National Endowment for the Arts provides evidence that museum attendance can slow decline in older adults, regardless of whether the participants create art or attend art (6). A 2021 American Alliance of Museums report, “Museums and Creative Aging: A Healthful Partnership,” details how diverse museums across the United States further enhance arts opportunities for older adults by offering specific creative aging programs, including museums from Puerto Rico, Kentucky, Alaska, Florida, and Mississippi (1).

Sometimes creative aging programs are developed by therapists such as music therapists, art therapists, or dance therapists, and the focus is on using the therapists’ art form to work on therapeutic goals and outcomes for patients in many settings (2, 3, 10–17). For example, music therapists may use active interventions such as instrument playing, developing a rock choir, iPad bands, or passive music interventions such as music-assisted relaxation, or song discussion groups to further enhance and support wellness in aging (10, 17), which is not dependent upon prior musical training (18, 19). One example of a program was conducted by González-Oja et al. 20, with 52 older adults residing in a nursing home. The researchers explored the effect of group music therapy interventions on older adults’ psychosocial and physical well-being. The group participated in singing, body percussion, movement to music, and instrument play interventions for 16 weeks. Results showed that older adults’ showed increases in their physical health and psychosocial well-being as measured by social interactions and creativity after participation in the music therapy program.

In the spring of 2020, many of these programs came to an immediate halt as the entire world experienced COVID- 19. Due to the rapid spreading of the virus, many arts-based community agencies in the United States closed their doors indefinitely. Senior living communities became a contained unit, and did not allow for many music therapists, or leaders of creative aging and lifelong learning programs to lead sessions for older adults. This necessary act at the beginning of the pandemic unfortunately negatively impacted older adults’ psychosocial well-being. The art of gathering together was something that was no longer safe. The act of making music together especially through singing in the same room could lead to a spread of the virus.

Virtual programming and telehealth were service delivery methods that existed prior to COVID-19, and some practitioners provided their services that way. However, as COVID-19 continued from the spring into summer, many practitioners began to develop special virtual programming to interact with older adults (21–23). New programs and programs that were once delivered in person, were now being offered through asynchronous and synchronous settings. Recent research in virtually delivered programs demonstrates that technology may be an effective platform for increasing accessibility for audiences to these experiences (24), with one intergenerational, music-based virtual study noting “the use of simple and free computer technology (e.g., Dropbox, tablets, smartphones) could be a promising vehicle for communicating, learning, and enhancing relationships between opposite generations” (11). One example of adapting in person sessions to telehealth was conducted by McAulay, Block, Booth, and Cowley (25). Forty-one older adults residing in a facility participated in 12 virtual group music therapy sessions across 6 weeks. Patient mood, engagement, and relaxation as responses to the interventions were measured for each older adult. Results for this study was mixed. While the majority of older adults showed improvements in mood, full or partial engagement, and relaxation, some older adults displayed no changed in mood or a negative mood, limited engagement, and no relaxation. There is still a lot to learn about providing asynchronous and synchronous virtual music sessions for older adults. Results from Wilhelm and Wilhelm’s survey (23) of music therapists showed that 64% of their respondents used teletherapy sessions, but only 48% would continue to do so after the pandemic. Results also showed a variety in the types of platforms used for sessions, and a variety of interventions used in session, with therapeutic singing, music listening, and music-cued reminiscing being the most common.

As humanities and health-based research in aging increases, organizations are responding, and they are especially considering the impacts of COVID-19 on older audiences. The American Alliance of Museums has called for museums to “invest in a diverse array of onsite and online programs that encourage healthy and active aging” and “foster new kinds of research and partnerships that can advance these goals” (1). Early in the pandemic, the International Council on Active Aging (ICAA) assembled a Senior Living Task Force and advocated for the increased use of technology and the recognition of multidimensional wellness (26). With these recommendations in mind and in response to the significant social isolation many older adults experienced during COVID-19, this paper will explore one such program that was developed between a global music museum and a university music therapy program for older adults in the Southwest. The purpose of this program was to explore the effect of a virtual creative aging program on older adults’ psychosocial well-being and engagement behaviors. The research questions are: (1) How does participation in a virtual creative aging program affect older adults’ psychosocial well-being? (2) How does participation in a virtual creative aging program affect older adults’ engagement behaviors? and (3) What are older adult’s perceptions of a virtual creative aging program?

## Materials and methods

### Project background

A global music museum and a music therapy program at a university in the Southwest began a formal partnership in early 2020 to engage board-certified graduate music therapy students in on-site music and memory care experiences. The partnership is unique because of the focus on cultural diversity. The museum is organized by galleries, each with a different geographic focus around the world. Each gallery hosts displays of instruments, music making accessories, and audio and visual clips of performers that are unique to that culture. Geographic galleries include: Africa, Asia, Europe, Latin America, the Middle East, and USA/Canada. The music therapist involved in the project has a research area in cultural diversity and aging, and taught classes on this topic at the time the partnership began.

The onset of the pandemic and subsequent closure of the museum delayed the launch of the program and encouraged organizers to consider new ways for sharing programming with older adult audiences. The professor of music therapy at the university and the curator of education at the museum (both principal investigators in this study) developed 30–45-min Zoom sessions that partnered museum gallery content with music therapy interventions. Between May 2020 and April 2021, the synchronous program reached over 550 senior participants through 22 sessions. This demand led the museum and the university partners to further collaborate on asynchronous video content to serve a larger audience. Organized and overseen by the principal investigators, the museum’s curatorial team of five subject-area experts and an upper division music therapy class composed of juniors and 1st year graduate equivalency students (*n* = 21) developed and filmed content.

### Museum content and music therapy interventions

Two video collections were produced, one for active seniors (senior wellness) and another for seniors with memory loss (memory care). Each collection contains five 15-min videos with an emphasis on content from various geographic locations, including USA, Europe, Latin America, Asia, Oceania, Africa, and the Middle East. Each video combines the museum content with music therapy activities and engages seniors in cognitive, physical, and psychosocial ways that explore specific geographic regions. For example, a video focused on West Africa includes content from the Sierra Leone and Liberia exhibits and musical content from Ella Jenkins’s “West African call-and-response” and the West African *kuku* drum pattern. Video content, musical selections, and music therapy focus are outlined in Tables 1, 2. The layout for each video was musical introduction, museum intervention 1, music therapy intervention 1, museum intervention 2, music therapy intervention 2, and musical outro.

**Table 1:** Gallery and music therapy focus for the memory care collection.

Gallery	Music and movement	Music therapy focus	Intervention types
Africa: Sierra Leone and Liberia	Ella Jenkins’s West African call-and-response and *kuku* drum and dance patterns	Foster short-term memory (cognitive), foster upper extremity movement (physical)	Singing, instrument, movement
Oceania: Polynesia	“E Te Iwi E” from New Zealand	Upper-body and bilateral movement (physical), sustained attention (cognitive)	Instrument, movement
Latin America: Mexico	“Cielito Lindo” and “Cumbia Pa′ Gozar” (Los Ángeles Azules)	Promote sustained attention (cognitive), promote gross motor movement across upper and lower extremities (physical)	Instrument, movement
USA/Canada: Chicago	12-bar blues and “Be Still” gospel hymn	Maintain sustained attention and foster short-term recall (cognitive), maintain gross motor movements across upper and lower extremities (physical), foster self-expression (psychosocial)	Singing, movement
Europe: Ireland and the UK	“All You Need Is Love” (The Beatles) and “Hall of Fame (The Script)	Divided attention in singing and moving (cognitive), retrieval of newly learned material (cognitive), upper extremity and gross motor movement (physical)	Instrument, movement

**Table 2:** Gallery and music therapy focus for the senior wellness collection.

Gallery	Music and movement	Music therapy focus	Intervention type
Middle East: Egypt, Turkey, and Israel	“Eich Efshar” (Jane Bordeaux)	Maintain memory recall (cognitive), self-expression through movement (psychosocial)	Instrument, movement
Asia: Taiwan and China	“Little Umbrella” (Chris Hung) and “Penghu Bay”	Maintain memory recall (cognitive), self-expression through movement (psychosocial)	Instrument, movement
Latin America: Caribbean	Puerto Rican *bomba* and “Three Little Birds” (Bob Marley and the Wailers)	Promote quality of life through learning new musical styles (psychosocial), promote divided attention through movement and singing (physical)	Instrument, movement
USA/Canada: Los Angeles	“My Wild Love” (The Doors) and “Superstition” (Stevie Wonder)	Foster retrieval of newly learned information (cognitive), foster and maintain gross motor skills (physical)	Instrument, movement
Europe: Ukraine	“*Shchedryk*” (“The Little Swallow”) and “*Yihav Kozak za Dunaj*” (“The Cossack Rode Beyond the Danube”)	Promote gross motor movement for upper and lower extremities (physical), promote self-expression through instrument playing and movement (psychosocial)	Instrument, movement

During the development of the museum gallery content, curatorial speakers considered how to engage the imagined audience through a balance of shared information, question prompts, and listening examples. Curators made efforts to simplify language and slow the rate of speech to accommodate participants experiencing memory loss. Video producers also ensured that camera movements and transitions were seamless and not abrupt. Questioning techniques encouraged the audience to stop or pause videos to promote further discussion. For example, a curator may say, “As you look at the exhibit, what objects stand out to you the most?” or “What do you notice?” followed by a wide camera angle of the whole exhibit. Curators also made efforts to encourage active participation during moments of listening (i.e., “As we listen to this upcoming clip, take note of the intricate rhythmic patterns the ensemble creates. See if you can find a steady beat on your lap”). The goal was to generate discussion, active listening, and personal or collective alignment with the material.

Music therapy interventions used in the videos were singing, movement, and instrument playing.

#### Singing

Interventions were coded as singing, if the primary goal was music engagement through singing. In these interventions music therapy students introduced songs from the region highlighted in the gallery. They taught the lyrics and pitches to the song and encouraged participants to sing along. There were two singing interventions that were labeled as singing + as the music therapy students added in instrument playing or moving after teaching the lyrics and pitches of the song.

#### Movement

Movement interventions featured traditional movements from the region sequenced together with a song, or students developed sequential movements that told the story of the song. For example, in the Latin America video from the Memory Care Collection, students taught older adults how to dance the cumbia. Whereas students in the Senior Wellness collection used the lyrics from the “Little Umbrella” in the Asia gallery, to develop sequential movements for the older adults.

#### Instrument

Interventions were coded as instruments if the primary task was making an instrument or learning rhythmic patterns on an instrument or using body percussion in a sequential pattern. Instrument making interventions featured music therapy students making rhythm instruments out of everyday items found in the home and encouraged older adults to engage in making their own rhythm instrument and then using them in the rest of the video session. Instrument play interventions featured students teaching rhythmic patterns and how to play instruments from the region highlighted in the gallery. Body percussion interventions featured music therapy students teaching older adults a series of body percussion such as stomping, clapping, tapping that could be used along with songs in the video.

### Participants

During the piloting phase of the project, 41 long-term care facilities from five US States (GA-3, PA-15, CO-2, WA-1, and AZ-20) received access to the program in exchange for feedback related to implementation and satisfaction. Three of these facilities, all located in the southwest, volunteered to participate in this current study (two skilled nursing facilities and one assisted living center). Life Enrichment staff shared general details about the video collections and evaluation with cognitively healthy long-term care residents and the family members/guardians of those residents with dementia. Residents were eligible to participate in the evaluation if they gave written consent (if they were deemed cognitively healthy by facility staff), or if their family member or legal guardian gave consent on their behalf over the telephone (for those with dementia) and the resident provided verbal assent before each activity session. A total of 27 cognitively healthy residents consented to participate at the beginning of the study and a total of 29 residents with dementia opted to participate, based on the consent of their family member or legal guardian and their continuing assent during the activity sessions.

### Study design and measures

A within-subject repeated measures design was used to investigate the impact of the creative aging program on older adults’ psychosocial well-being and engagement behaviors. A variety of measures were used at pre-program, mid-program, and post-program. Psychosocial well-being for cognitively healthy older adults was defined as quality of life, engagement in meaningful activities and social isolation. Whereas, psychosocial well-being for older adults diagnosed with dementia was defined as quality of life.

### Psychosocial well-being for cognitively healthy older adults

#### Quality of Life

Healthy older adult participants’ (*n* = 27) quality of life was examined with a standardized measure, administered via paper-and-pencil, at three time points (pre-program, mid-program, and post-program). The quality of life of cognitively healthy residents was measured with the Multicultural Quality of Life Index, which assesses 10 dimensions of subjective quality of life such as physical well-being and spiritual fulfillment (27). Each domain is rated with a single item on a scale of 1–10, with 1 indicating ‘Poor’ and 10 indicating ‘Excellent’ present quality of life. Two domains which typically do not apply to long-term care residents (occupational functioning and community and services support) were eliminated, for a total of 8 items. All items were summed and divided by the number completed (1–8) for a total Quality of Life score, with higher scores indicating greater quality of life. Cognitively healthy residents completed the questionnaires independently, unless they had physical or visual impairments which impeded their ability to do so; in this case, the researcher or Life Enrichment (Activities) staff offered assistance by reading the questions aloud and marking down the residents’ response.

#### Engagement in meaningful activities

Cognitively healthy residents also responded to questions about their activities, utilizing the Engagement in Meaningful Activities Survey (EMAS) (28) at three time points (pre-program, mid-program, and post-program). The EMAS is a 12-item scale which measures impressions of the meaningfulness of daily activities such as “The activities I do express my creativity” and “give me a sense of satisfaction.” Items are measured on a scale of 1–4, with 1 indicating ‘Rarely’ and 4 indicating ‘Always.’ All items were summed, with higher scores indicating greater meaningfulness of activities.

#### Social isolation

Cognitively healthy residents also responded to questions about their social relationships, utilizing the Patient-Reported Outcomes Measurement Information System (PROMIS) Social Isolation Short Form-4a (29) at 3 time points (pre-program, mid-program, and post-program). The measure consists of 4 items about social health such as “I feel left out” or “I feel that people barely know me.” The items are rated on a scale from 1 to 5, with 1 indicating ‘Never’ and 5 indicating ‘Always.’ All items were summed, with higher scores indicating greater feelings of social isolation.

### Psychosocial well-being for older adults with dementia

#### Quality of life

The quality of life of older adult participants with dementia (*n* = 29) were examined at three time points (pre-program, mid-program, and post-program) utilizing the Quality of Life in Late-Stage Dementia (QUALID) scale (30). The QUALID consists of 11 items which ask the respondent to rate observed resident behaviors, such as crying or appearing physically uncomfortable, over the past week. Each item is rated according to the amount of time that the behavior has been displayed, such as ‘rarely or never’ to ‘almost always.’ After reverse-scoring items for consistency in amount of time, all items were summed, with higher scores indicating greater quality of life.

### Resident music engagement

Engagement with the video collections for both participant groups was measured by an adapted version of the Music in Dementia Assessment Scale (MIDAS) observation tool (31). The MIDAS allows trained raters to observe and quantify participant behaviors at 4 points during each session: 15 min before the session, halfway through the video interventions (after the introduction, museum intervention 1 and music therapy intervention 1), at the conclusion of the video program (after museum intervention 2, music therapy intervention 2, and outro), and 15 min after a video. These behaviors include participant interest (posture, facial expressions, and/or animations that demonstrated interest), response (body movements, eye contact, and/or musical interactions that were related to the intervention/video segment), initiation (conversation engagement and/or reminiscing that occurred while watching the video segment), involvement (engagement and/or enthusiasm toward the intervention), and enjoyment (smiling, laughing, and/or relaxation in response to the video). For the current study, the MIDAS was adapted for ease of use and interpretation by converting to a 5-point Likert scale from a Visual Analog Scale, with 1 being little to none and 5 being very high. In addition, the adapted MIDAS was applied to the resident group as a whole, rather than each individual resident. The study researchers (3rd author and an additional collaborator) served as data collectors, and were trained by the first two authors to promote consistency across ratings. At the end of each activity session, residents from both groups were also asked the following open-ended questions about their impressions: “What did you enjoy in today’s session?” and “What do you wish there were more of?”

### Procedure

Life Enrichment (Activities) staff from the three communities led five music-focused activities sessions for residents, centered around the video collections, over an eight-week period. Each session ranged from 30 to 60 min. Two communities opted to run separate sessions for cognitively healthy residents (using the Senior Wellness Collection) and those with dementia (using the Memory Care Collection), while one community combined these resident groups into a single, weekly session using videos from both collections. Life Enrichment staff were supplied with electronic access to all materials: the Senior Wellness and Memory Care video collections, viewing guides to assist with effective facilitation of group sessions, and individual video activity guides with suggestions for how to expand upon the learning and engagement intended in the video collection. Project developers and researchers also hosted a one-hour introductory meeting to discuss strategies for implementation and answer any questions.

### Data analysis

#### Psychosocial well-being measures

Participation at each data collection time point (pre-test, midpoint, post-test) varied due to resident discharge from the facility, health changes that prevented attendance at the session (such as a COVID-19 infection) or death. To account for missing data, the last data point collected from each resident (midpoint or post-test; *N* = range of 16–19, depending on the measure) was compared to pre-test scores using repeated measures *t*-tests to statistically analyze change in resident outcomes during the study period.

#### Musical engagement measures

Descriptive statistics was used to analyze which engagement behaviors were demonstrated by older adults as they participated in the virtual programming. A Mann Whitney *U* test was used to analyze how cognitively healthy older adults and older adults with dementia were engaged with content. The five engagement behaviors were: interest, response, initiation, involvement, and enjoyment. Descriptive statistics was also used to analyze which intervention (singing, movement, playing instruments) elicited more engagement behaviors. The data, group MIDAS scores from 4 test points (15 min before, halfway through the video interventions, at the conclusion of the video program, and 15 min after a video) were consolidated by totaling and averaging the data to provide total and average scores for each engagement behavior. Similarly, engagement behaviors scores were totaled and averaged per intervention for comparison.

#### Perception of creative aging program

The collective responses to the MIDAS questions (“What did you enjoy in today’s session?” and “What do you wish there were more of?”) were analyzed using a word frequency text analyzer and then converted into a word cloud using a Microsoft add-in program to illustrate emerging themes. Common words like articles were not included, and researchers reviewed data to ensure consistency of responses before analyzing. For example, words like dance and dancing were both coded as dancing to accurately reflect participant interest in the visualization.

## Results

### Psychosocial well-being for cognitively healthy older adults

Cognitively healthy residents attended an average of 3 of the 5 activity sessions; 56% of these residents attended four or five sessions (*N* = 14/25). Psychosocial well-being for cognitively healthy older adults was measured using standardized tests for their quality of life, engagement in meaningful activities, and social isolation. Repeated measures *t*-tests comparing the pre-test to the last data point collected from each cognitively healthy resident (*N* = 19) are reported in Table 3. Correlations between session attendance and psychosocial well-being can be found in Table 4. Overall, the number of sessions attended was not significantly related to the outcomes of interest.

**Table 3:** Pre-test and last test comparison of psychosocial well-being for cognitively healthy older adults.

Domain	Variable	Pre-test Mean (SD)	Last test Mean (SD)	Statistical significance
Quality of life	Overall quality of life (summary score)	3.80 (0.59)	3.75 (0.77)	*t*(18) = −0.29, *p* ≤ 0.77, *d* = 0.07
	Physical wellness	3.16 (0.90)	3.32 (1.06)	*t*(18) = 0.68, *p* ≤ 0.51, *d* = 0.16
	**Psychological/emotional wellness**	**3.56 (0.86)**	**3.94 (0.94)**	***t*** **(17) = 2.12, ***p*** ≤ 0.05, ***d*** = 0.50**
	Self-care/independent functioning	3.69 (0.95)	3.63 (1.15)	*t*(15) = −0.24, *p* ≤ 0.82, *d* = 0.06
	Interpersonal functioning	4.29 (0.69)	4.12 (0.78)	*t*(16) = −0.72, *p* ≤ 0.48, *d* = 0.17
	Social–emotional support	3.94 (0.83)	4.24 (0.83)	*t*(16) = 1.23, *p* ≤ 0.24, *d* = 0.30
	Personal fulfillment	3.82 (0.81)	3.47 (1.07)	*t*(16) = −1.46, *p* ≤ 0.16, *d* = 0.35
	Spiritual fulfillment	4.00 (1.03)	4.17 (0.86)	*t*(17) = 0.62, *p* ≤ 0.55, *d* = 0.14
	General quality of life	3.78 (0.94)	3.39 (1.33)	*t*(17) = −1.59, *p* ≤ 0.13, *d* = 0.38
Meaningful engagement in activities	Overall meaningful engagement in activities	42.68 (9.14)	43.74 (10.42)	*t*(18) = 0.63, *p* ≤ 0.54, *d* = 0.14
	The activities I do…help me take care of myself	3.78 (1.22)	3.72 (1.32)	*t*(17) = −0.18, *p* ≤ 0.86, *d* = 0.04
	…reflect the kind of person I am	3.78 (0.88)	3.72 (1.13)	*t*(17) = −0.27, *p* ≤ 0.79, *d* = 0.06
	…express my creativity	3.44 (0.98)	3.61 (1.20)	*t*(17) = 0.59, *p* ≤ 0.56, *d* = 0.14
	…gives me a sense of accomplishment	3.72 (1.32)	3.61 (0.92)	*t*(17) = −0.32, *p* ≤ 0.76, *d* = 0.07
	…contribute to my feeling competent	3.90 (0.88)	3.58 (1.35)	*t*(18) = −1.24, *p* ≤ 0.23, *d* = 0.28
	…are valued by other people	3.47 (1.02)	3.26 (1.10)	*t*(18) = −0.68, *p* ≤ 0.51, *d* = 0.16
	…help other people	3.44 (1.25)	3.33 (1.19)	*t*(17) = −0.33, *p* ≤ 0.75, *d* = 0.07
	…give me pleasure	4.11 (0.83)	4.39 (0.85)	*t*(17) = 1.23, *p* ≤ 0.24, *d* = 0.29
	…give me a feeling of control	3.28 (1.13)	3.72 (1.18)	*t*(17) = 1.19, *p* ≤ 0.25, *d* = 0.28
	…help express my personal values	3.83 (1.15)	3.44 (1.38)	*t*(17) = −1.16, *p* ≤ 0.26, *d* = 0.27
	…gives me a sense of satisfaction	3.90 (0.94)	4.05 (1.03)	*t*(18) = 0.77, *p* ≤ 0.45, *d* = 0.18
	**…have the right amount of challenge**	**3.42 (1.07)**	**3.95 (0.85)**	***t*** **(18) = 2.54, ***p*** ≤ 0.02, ***d*** = 0.58**
Social isolation	**Overall feelings of social isolation**	**6.63 (2.77)**	**9.37 (4.96)**	***t*** **(18) = 2.62, ***p*** ≤ 0.02, ***d*** = 0.60**
	**I feel…left out**	**1.67 (0.84)**	**2.72 (1.53)**	***t*** **(17) = 2.89, ***p*** ≤ 0.01, ***d*** = 0.68**
	…that people barely know me	1.71 (1.05)	2.47 (1.55)	*t*(16) = 2.07, *p* ≤ 0.06, *d* = 0.50
	…isolated from others	1.44 (0.78)	1.83 (1.25)	*t*(17) = 1.59, *p* ≤ 0.13, *d* = 0.38
	…people are around me but not with me	2.42 (1.35)	2.16 (1.21)	*t*(18) = 0.68, *p* ≤ 0.51, *d* = 0.16

Bold text indicates a statistically significant difference between pre-test and last data point collected at *p* ≤ 0.05.

**Table 4:** Correlation between number of activity sessions attended and psychosocial well-being outcomes for cognitively healthy older adults (*N* = 25).

	1	2	3	4
Number of sessions attended	--			
Quality of life	0.22	**--**		
Meaningful engagement in activities	0.05	**0.72**	**--**	
Social isolation	0.16	−0.15	0.26	**--**

Bold text indicates a statistically significant relationship at *p* ≤ 0.05.

Residents reported overall good quality of life at the pre-test and showed little change in overall quality of life at the last time point. Yet, when quality of life domains were examined separately, residents did show a statistically significant increase in psychological/emotional wellness after the music therapy intervention (*t*(17) = 2.12, *p* ≤ 0.05, *d* = 0.50).

Cognitively healthy residents did not show a statistically significant increase in meaningful engagement in activities after the music therapy intervention. However, across individual engagement items, there was a statistically significant increase in residents agreeing that their activities have the right amount of challenge (*t*(18) = 2.54, *p* ≤ 0.02, *d* = 0.58). While not statistically significant, there were also mean increases in residents agreeing that their activities help express their creativity, give them pleasure, give them a feeling of control, and a sense of satisfaction.

Cognitively healthy residents reported an increase in overall social isolation after the music therapy intervention (*t*(18) = 2.62, *p* ≤ 0.02, *d* = 0.60). Examining individual items showed a statistically significant increase in feeling left out between pre-test and last data point collected (*t*(17) = 2.89, *p* ≤ 0.01, *d* = 0.68). There were also mean increases in residents feeling that people barely know them, feeling isolated from others, and feeling that people are around them but not with them, although not statistically significant.

### Psychosocial well-being for older adults diagnosed with dementia

For older adults in memory care, residents also attended an average of 3 of the 5 activity sessions; 58% of these residents attended four or five sessions (*N* = 14/24). Caregiver-reported quality of life of these residents is reported in Table 5. The correlation between the number of sessions attended and caregiver-reported quality of life was not statistically significant (*r*(23) = −0.26, *p* ≤ 0.22).

**Table 5:** Pre-test and last-test comparison of psychosocial well-being for older adults with dementia.

Domain	Variable	Pre-test Mean (SD)	Last test Mean (SD)	Statistical significance
Quality of life	Overall quality of life (summary score)	27.57 (4.79)	26.83 (3.89)	*t*(22) = 0.89, *p* ≤ 0.38, *d* = 0.19
	Resident smiles	4.23 (1.17)	4.48 (0.85)	*t*(22) = −1.03, *p* ≤ 0.31, *d* = 0.21
	Appears sad	1.96 (1.15)	1.61 (0.94)	*t*(22) = 1.40, *p* ≤ 0.18, *d* = 0.29
	Cries	1.31 (0.70)	1.22 (0.67)	*t*(22) = 0.53, *p* ≤ 0.60, *d* = 0.11
	Appears unhappy or in pain (facial expression of discomfort)	1.96 (0.98)	1.78 (0.90)	*t*(22) = 1.00, *p* ≤ 0.34, *d* = 0.21
	**Appears physically uncomfortable**	**2.26 (1.14)**	**1.48 (0.90)**	***t*** **(22) = 3.95, ***p*** ≤ 0.001, ***d*** =** 0.82
	Makes statements or sounds suggesting discontent, unhappiness, or discomfort	2.00 (1.24)	1.78 (1.28)	*t*(22) = 0.76, *p* ≤ 0.46, *d* = 0.16
	**Appears irritable or aggressive**	**1.61 (0.84)**	**1.26 (0.45)**	***t*** **(22) = 2.34, ***p*** ≤ 0.03, ***d*** = 0.49**
	Enjoys eating	4.39 (1.03)	4.61 (0.66)	*t*(22) = 1.00, *p* ≤ 0.34, *d* = 0.30
	Enjoy touching others or being touched	3.86 (0.83)	4.18 (0.85)	*t*(21) = −1.91, *p* ≤ 0.07, *d* = 0.41
	**Enjoy interacting or being with others**	**4.17 (0.78)**	**4.48 (0.67)**	***t*** **(22) = −2.30, ***p*** ≤ 0.03, ***d*** = 0.48**

Bold text indicates a statistically significant difference between pre-test and last data point collected at *p* ≤ 0.05.

Similar to the cognitively healthy residents, there was no change in overall quality of life for residents in memory care. Yet, investigation of separate quality of life domains showed a statistically significant decrease in appearing physically uncomfortable (*t*(22) = 3.95, *p* ≤ 0.001, *d* = 0.82) and being irritable or aggressive (*t*(22) = 2.34, *p* ≤ 0.03, *d* = 0.49). In addition, there was a statistically significant increase in enjoying interacting or being with others (*t*(22) = −2.30, *p* ≤ 0.03, *d* = 0.48).

### Engagement behaviors

Engagement behaviors for all older adult participants were measured through observation of participants at four points during each session: (a) 15-min prior to the session, (b) halfway through the session (after the intro, museum intervention 1 and music therapy intervention), (c) at the end of session (after museum intervention 2, music therapy intervention 2, and outro), and (d) 15-min post session. Engagement behaviors were defined as interest, response, initiation, involvement, and enjoyment. A Mann–Whitney *U* test was conducted on weekly data of participants an apostrophe to participants’ engagement behavior to determine if cognitively healthy older adults and older adults with memory loss were similarly engaged. Results revealed that cognitively health older adults demonstrated significantly more engagement behaviors following the virtual program than older adults with dementia when participating in the sessions (*z* = 2.51, *p* < 0.05). See Table 6 for total and average engagement behavior scores for all older adults.

**Table 6:** Total and average engagement behaviors across all sessions.

Category	Interest	Response	Initiation	Involvement	Enjoyment
Total engagement behaviors cog. healthy O.A.	121	109	105	103	103
Average engagement behaviors cog. healthy O.A.	4.65	4.19	4.04	3.96	3.96
Total engagement behaviors O.A. w. dementia	91	79	67	75	69
Older adults diagnosed with dementia	3.79	3.29	2.79	3.13	2.88

Cognitively healthy older adults’ top three engagement behaviors while participating in virtual creative aging programming were (1) interest (*M* = 4.65), (2) response (*M* = 4.19), and (3) initiation (*M* = 4.04). Whereas older adults diagnosed with dementia top three engagement behaviors while participating in virtual creative aging programming were (1) interest (*M* = 3.79), (2) response (*M* = 3.29), and (3) involvement (*M* = 3.13).

A descriptive analysis was conducted to determine if more engagement behaviors for older adults occurred following specific interventions. Results showed that cognitively healthy older adults displayed more engagement behaviors during instrument interventions (*M* = 4.28) compared to older adults diagnosed with dementia (*M* = 3.22). Whereas older adults with dementia demonstrated the most engagement behaviors during singing interventions. See Table 7 for more details.

**Table 7:** Average engagement behaviors across all interventions.

Category	Instrument play	Movement	Singing +
Cognitively healthy older adults	4.28	4.15	No interventions during this video collection
Older adults diagnosed with dementia	3.22	2.91	3.47

### Perception of creative aging program

**Figure 1:**
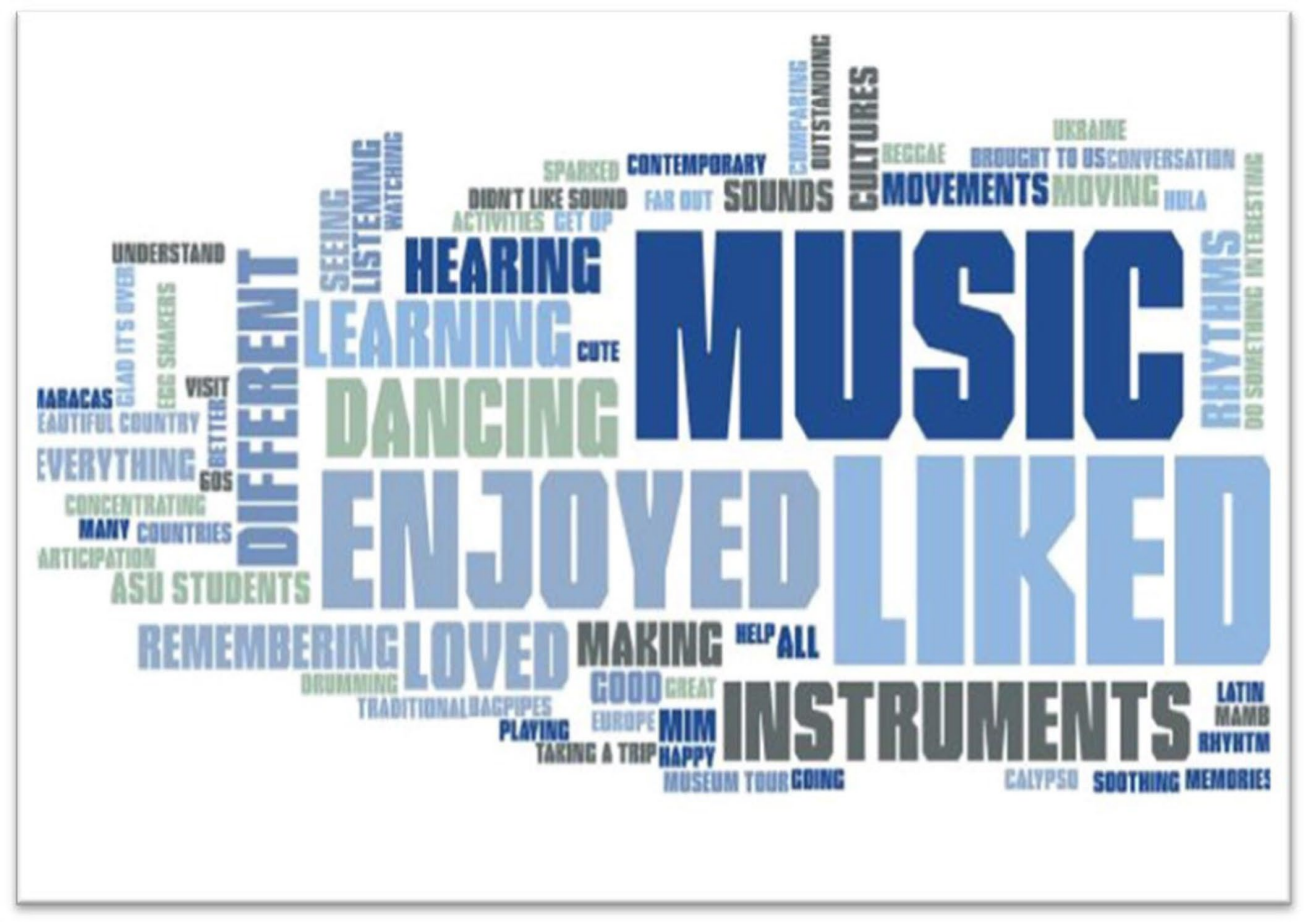
Participant responses to “what did you enjoy about today’s session.”

**Figure 2:**
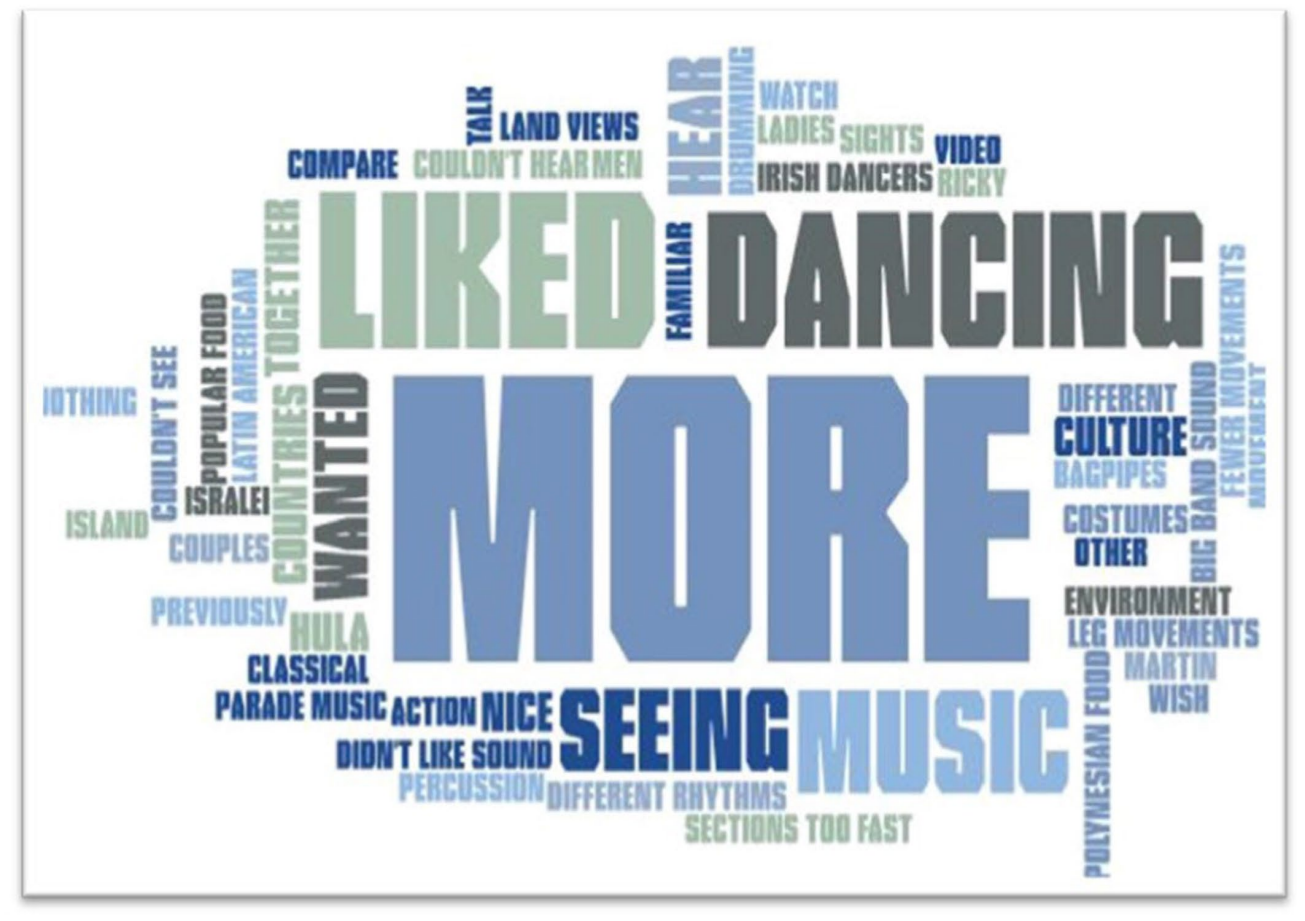
Participant responses to “what do you wish would have been included in today’s session.”

At the conclusion of each video program, participants were asked two open-ended questions as a part of the MIDAS assessment: “what did you enjoy in today’s session” and “what do you wish there were more of.” The word frequency text analysis demonstrates a positive perception of the program. In response to “what did you enjoy about today’s session,” participants used the words “music” and “liked” 17 times and “enjoyed” appeared 11 times. The words “dancing,” “instruments,” “learning,” and “loved” each appeared 5–7 times. In response to “what do you wish would have been included in today’s session,” participants noted they wanted “more” 25 times and “liked” appeared 13 times. Other frequent words included “dancing” (11), “music” (7), “seeing” (6), and “hearing” (4). The full word frequency analysis is demonstrated through a word cloud in Figures 1, 2.

## Discussion

The purpose of this study was to explore the effect of a virtual creative aging program on older adults’ psychosocial well-being and engagement behaviors. Older adults participated in a five-session program across 8 weeks that was delivered in group settings with the Life Enrichment staff at their facility. A total of 56 older adults defined as either cognitively healthy (*n* = 27) engaged with videos from the Senior Wellness Collection (Asia, Europe, Latin America, Middle East and USA/Canada) or diagnosed with dementia (*n* = 29) engaged with videos from the Memory Care Collection (Africa, Europe, Latin America, Oceania and USA/Canada).

Cognitively healthy older adults who participated in the creative aging program showed an increase in their emotional well-being over 8 weeks. This aligns with other studies of older adult music participation and listening, which suggest an improvement in mental well-being and mood (32). In contrast, cognitively healthy older adults showed no change in their meaningful engagement in activities. This is counter to some previous studies which found that participation in therapeutic programs incorporating music was associated with greater engagement in meaningful activities (33). Similarly, other studies have shown that music participation is related to a greater sense of accomplishment, feelings of competence, and purpose, similar to the concept of meaningful engagement (34).

Surprisingly, cognitively healthy older adults showed an increase in their social isolation over the 8-week period. This is in contrast to many studies which have found improved social connection associated with music participation (35, 36). One possible explanation for the increased social isolation seen in the current study may lie in the external challenges that the participating facilities faced during the creative aging program. All three experienced COVID-19 outbreaks during the intervention (roughly around Week 4) where the creative aging program had to be paused, and older adult residents were quarantined to their rooms to prevent further spread of infection. It is possible that the isolating experience of the COVID-19 mitigation protocols influenced the older adults’ responses on the questionnaires and potentially had a greater impact on the older adults’ perceived social isolation than the creative aging program was able to attenuate.

Older adults with dementia who participated in the creative aging program showed a decrease in physical discomfort and irritation and an increase in enjoying being with others, as rated by their caregivers. Other studies have similarly found that music interventions can reduce agitation and aggression among those with dementia as well as improve social engagement (37–39).

Both cognitively healthy older adults and older adults diagnosed with dementia exhibited engagement behaviors while participating in the program. The engagement behaviors for all older adults were similar and different. Both cognitively healthy older adults and those diagnosed with dementia demonstrated interest (facial expression and posture responses to the interventions in the video) and response (body movement, eye contact, and musical interaction with the leaders in the videos) as their top engagement behaviors when interacting with the creative aging program. However, cognitively healthy older adults demonstrated more initiation behaviors (conversation engagement and/or reminiscing that occurred while watching the video segment) compared to older adults diagnosed with dementia who demonstrated more involvement responses (engagement and/or enthusiasm toward the intervention). This makes sense based on their cognitive ability. It was probably easier for the cognitively healthy older adults to engage in reminiscing experiences during the session compared to older adults with dementia. As one progresses through the illness, cognitive discussions can become difficult, and it may be easier to engage through participating in interventions without an elongated discussion (15).

While older adult participants responded to all interventions with engagement behaviors, instrument interventions were most engaging for cognitively healthy older adults. Singing interventions were most engaging for older adults with dementia, whereas movement interventions were less engaging for older adults with dementia. Perhaps this was due to the number of sequential patterns involved in the movements which could be considered a cognitively high task as there were no visual or written cues on the screen.

The positive perception of the program by participants aligns with the recent research that virtually delivered programs are an effective platform to increase accessibility and enhance communication (11, 24). Furthermore, the qualitative data suggested a high level of engagement with the material. Pairing lifelong learning content with music-based interventions may provide a favorable balance of new information with active interventions. For museum practitioners, this signals a continued inclusion of museum content within creative aging programs.

### Limitations

There are a few limitations to this study’s findings which should be acknowledged. The evaluation study conducted was observational, rather than experimental. Accordingly, there was no control group who did not receive the music intervention, which limits our ability to assert that the intervention caused the outcomes seen. However, within-subjects designs have the advantage of requiring fewer participants to demonstrate effects and reduce the impact of variability due to individual participant differences. Nevertheless, a relatively small sample of older adult residents participated in the research study and completed questionnaires, although additional residents were interested in the creative aging program and often sat in on the sessions. This could have limited our power to detect statistically significant differences between the pre-test and last point of data collected, especially given that the size of most effects were small (i.e., Cohen’s *d* less than 0.20). Next, COVID-19-related breaks in the sessions, as previously discussed, may have attenuated the impact of the creative aging program. Furthermore, some of the survey items focused on domains which are typically impacted by aging and institutional residence (e.g., ability for independent functioning, feelings of control, feelings of competence) which could have influenced the older adults’ responses and partially explain the lack of change in these domains. Another limitation is with the interventions. Many interventions in the wellness collection did not include a singing-only intervention. In future studies, it would be interesting to have the same types of interventions in each collection for comparison. Similarly, the creative aging program contains unique gallery tours, curator-posed engagement questions, and broad-based music therapy foci, which may not translate to other music-focused programming. As a result, this study’s findings may be less generalizable to other music interventions.

### Implications and next steps

Overall, the results of this study show promise for creative aging programs to be delivered virtually. While the original videos were 15 minutes, the feedback from participants on wanting more, and the ways that the Life Enrichment staff directors engaged with the videos (pausing, asking additional questions, etc.), suggests that video content could be longer. Perhaps the video content could be expanded to 30 minutes or more and could include references such as “pause here for reflection” in the video, and provide repetition of content or return to content provided earlier in the video, especially in the memory care collection. Another next step would be to include manipulatives that could be sent to the facilities or a list of manipulatives that staff could order for the session. For example, if someone on the video was talking about the texture of an instrument, it could enhance the experience if the leader at the facility could pass around the texture to connect the participants to the video even more.

## Data availability statement

The raw data supporting the conclusions of this article will be made available by the authors, without undue reservation.

## Ethics statement

The studies involving humans were approved by Arizona State University Institutions Review Board. The studies were conducted in accordance with the local legislation and institutional requirements. Written informed consent for participation in this study was provided by the participants' legal guardians/next of kin. Written informed consent was obtained from the individual(s) for the publication of any potentially identifiable images or data included in this article.

## Author contributions

MB: Conceptualization, Methodology, Investigation, Formal Analysis, Writing - Original draft. KP: Conceptualization, Methodology, Funding Acquisition, Investigation, Formal Analysis, Writing - Original draft. TLM: Project administration, Investigation, Formal Analysis, Writing - Original draft.
